# Sociodemographic characteristics of pediatric patients with vascular malformations: Results of a single site study

**DOI:** 10.3389/fped.2023.1078611

**Published:** 2023-02-16

**Authors:** Michael Mohnasky, Jennifer Brondon, Sang Yub Lee, Kyung Rae Kim

**Affiliations:** ^1^ School of Medicine, University of North Carolina, Chapel Hill, NC, United States; ^2^ Department of Pediatrics, University of North Carolina, Chapel Hill, NC, United States; ^3^ Department of Radiology, Samsung Medical Center, Seoul, South Korea; ^4^ Department of Radiology, University of North Carolina, Chapel Hill, NC, United States

**Keywords:** vascular malformations, healthcare disparities, pediatrics, race, sex, insurance coverage, ethnicity, interventional radiology

## Abstract

Vascular malformations, the abnormal development of blood vessels, are a rare set of congenital anomalies. The sociodemographic factors associated with vascular malformations in pediatric patients are poorly understood. This study examined sociodemographic factors of 352 patients presenting to a single vascular anomaly center from July 2019 to September 2022. Characteristics such as race, ethnicity, sex, age at presentation, degree of urbanization, and insurance status were recorded. This data was analyzed by comparing the different types of vascular malformations, including arteriovenous malformation, capillary malformation, venous malformation (VM), lymphatic malformation (LM), lymphedema, and overgrowth syndrome. Patients were primarily white, not Hispanic or Latino, female, had private health insurance, and were from the most urban setting. No differences in sociodemographic factors were found among the different vascular malformations except patients with VM presented at a later age than patients with LM or overgrowth syndrome. This study provides novel insight into the sociodemographic factors of pediatric patients presenting with vascular malformations and indicates a need for their improved recognition for the timely initiation of treatment.

## Introduction

Vascular anomalies are a broad spectrum of pathologies that consist of two broad categories: vascular tumors and vascular malformations (1). Vascular malformations are further divided into different categories depending on which portion of the vascular or lymphatic system is impacted and if they are a component of an underlying syndrome (2). Vascular malformations are thought to be present at birth due to impaired vascular or lymphatic morphogenesis in early development but may not become symptomatic until later in life, if ever (3). Vascular malformations in childhood may present a spectrum of morbidity, from cosmetic concerns to CNS involvement and systemic coagulopathy (3, 4). Vascular tumors, however, are caused by endothelial cell proliferation and categorized by their invasive potential. Though they may sometimes be confused with vascular malformations based on mutual rarity and symptomology, they represent a different disease process entirely (2). Despite significant research into the causes and treatments of vascular malformations, less is known about the diversity of pediatric patients presenting with these conditions.

Much research has explored how various sociodemographic factors interact with different diseases revealing disparities in prevalence and outcome. For rare diseases such as vascular malformations, difficulty receiving an accurate diagnosis is an example of how sociodemographic factors may intersect with disease and cause disparities. Genetic testing modalities are increasingly improving diagnostic ability for rare diseases. However, studies have shown that underrepresented minorities, including children, are less likely to receive genetic testing delaying or prohibiting a diagnosis from being made (5, 6). Also, one's ability to access expertise care may lead to an incorrect diagnosis. Vascular malformations are often misdiagnosed, delaying access to appropriate care and potentially resulting in improper treatment (7, 8). Expertise care needed for correct diagnosis and management may be inaccessible for patients and their families who live outside of the range of specialized vascular anomaly centers and lack the financial resources to travel long distances or miss work to do so (8). Highlighting disparities such as these can inform public health policy and practice to reduce the effect of sociodemographic characteristics on health.

Because of the rare nature of vascular malformations, it has been challenging to characterize sociodemographic factors of pediatric patients with these conditions. Without a comprehensive understanding of these factors, it is difficult to determine if and how these factors are associated with the development of vascular malformations or a delay in diagnosis or treatment. The purpose of this study was to examine the characteristics of a large population of pediatric patients with vascular malformations and investigate whether differences exist in sociodemographic factors.

## Methods

### Study design and participants

The institutional review of board approved this study and waived the need for informed consent. This is a cross-sectional study of 352 patients under the age of 18 presenting with vascular malformations at a single vascular anomaly center from July 2019 to September 2022. This vascular anomaly center is an academic institution outpatient center located in a suburban region in the Southern United States. The center is composed of a broad multidisciplinary team of approximately 40 providers spearheaded by pediatric interventional radiologists, pediatric dermatologists, and pediatric hematologists. Data was gathered regarding patient diagnosis and sociodemographic factors *via* chart review of the electronic medical record (EMR).

### Diagnosis categories

2018 ISSVA classification was used to categorize patient groups (1). Vascular malformations were categorized as arteriovenous malformation (AVM), capillary malformation (CM), venous malformation (VM), lymphatic malformation (LM), lymphedema, and overgrowth syndrome. Overgrowth syndrome included specific diagnoses of Klippel-Trenaunay syndrome (KTS), Congenital Lipomatous Overgrowth, Vascular Malformations, Epidermal Nevis, Spinal/Skeletal Anomalies/Scoliosis syndrome (CLOVES), Diffuse Capillary Malformation with Overgrowth (DCMO), Parkes Weber syndrome (PWS), PTEN hamartoma of the soft tissue (PHOST), and PIK3CA-Related Overgrowth Spectrum (PROS) other than KTS or CLOVES. Patients with isolated CM with no combined overgrowth syndrome or other vascular malformation were categorized into the CM group. Five patients had multiple vascular malformations that were not a part of a syndrome (VM with CM) and were classified as VM to reduce group complexity and because of the relative severity of the conditions. Patients who presented with vascular tumors were excluded from the study to narrow the scope of the study and reduce complexity.

### Demographic variables

Data was gathered from the electronic medical record regarding race, ethnicity, sex, age at presentation, insurance status, and residential information captured by rural-urban commuting area (RUCA) codes. Race was categorized as white, black or African American, Asian, American Indian or Alaskan Native, Other, or unknown if no data was available. Patients who identified as more than one race were coded as the race that is assigned in the EMR which may be one of their multiple races or “Other”. Insurance status was categorized as private, Medicaid, military, state-health plan, or self-pay. RUCA codes were gathered from the 2010 US census, and patient address was used to assign each patient a particular RUCA code (9). RUCA codes ranged from 1 to 10 and represent a continuum of the most urban setting <1> to the most rural setting <10>.

### Statistical analysis

Statistical analyses were conducted to assess whether relationships existed for a particular sociodemographic characteristic and a vascular malformation. All statistical analyses were performed using MedCalc statistical software version 20.015 (MedCalc Software Ltd, Ostend, Belgium). The *χ*^2^-test was used to test for differences among race, ethnicity, sex, and insurance status for each vascular malformation. The Shapiro–Wilk test was used to test for normality in comparing the mean age at presentation for each type of vascular malformation. Once normality was confirmed, the ANOVA test was used to test for significant differences among the groups, and the Tukey Kramer test was used to determine between which groups specifically the difference existed. *p* < 0.05 was used to determine statistical significance.

## Results

### Overall patient characteristics

A total of 352 patients presented with vascular malformations. Two hundred twenty (62.5%) were white, 286 (81.3%) were not Hispanic or Latino, and 205 (58.2%) were female. One hundred fifty-three (43.5%) had private insurance, 145 (41.2%) had Medicaid, while the rest had other forms of insurance. The average age of onset was 8.17 years (95% CI: 7.56 years to 8.78 years). The most common vascular malformations were VM (144 patients; 40.9%) and LM (112 patients; 31.8%). The most common diagnosis within the overgrowth syndromes was KTS (17 patients, 35.4%) (Table 1).

**Table 1:** Demographic characteristics and breakdown of vascular malformations.

Demographics	Patients (*n* = 352)
Female	205 (58.2%)
Age in years (95% CI)	8.15 (7.55 to 8.74)
**Race**	
White	220 (62.5%)
Black or African American	54 (15.3%)
Asian	8 (2.27%)
American Indian or Alaskan Native	5 (1.42%)
Other	58 (16.5%)
Unknown	7 (1.99%)
**Ethnicity**	
Not Hispanic or Latino	286 (81.3%)
Hispanic or Latino	61 (17.3%)
American Indian or Alaskan Native	1 (0.284%)
Unknown	4 (1.14%)
**Insurance**	
Private	153 (43.5%)
Medicaid	145 (41.2%)
Military	26 (7.39%)
State-health plan	9 (2.56%)
Self-pay	19 (5.40%)
**Vascular malformation type**	
Arteriovenous malformation	25 (7.10%)
Capillary malformation^a^	5 (1.42%)
Venous malformation	144 (40.9%)
Lymphatic malformation	112 (31.8%)
Lymphedema	18 (5.11%)
Overgrowth syndrome	48 (13.6%)
**Overgrowth syndrome type**	
KTS	17 (35.4%)
PROS^b^	14 (29.2%)
DCMO	8 (16.7%)
CLOVES	6 (12.5%)
PWS	2 (4.2%)
PHOST	1 (2.1%)

^a^ Includes only isolated CM with no combined overgrowth syndrome or other vascular malformation.

^b^ PROS group includes all PROS diseases other than KTS and CLOVES.

**Figure 1:**
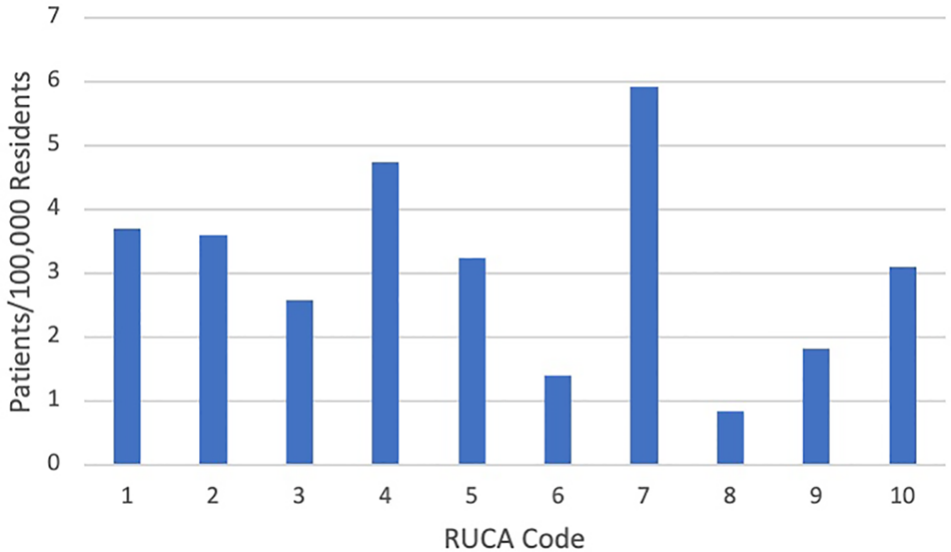
Proportion of vascular malformation patients by RUCA code.

One hundred ninety-eight (59.4%) fell within RUCA code 1, representing the most urban setting. Fifteen (4.26%) fell within RUCA code 7, which represents small-town regions, but this code had the most patients relative to the total population of that RUCA code (5.91 patients per 100,000 residents) (Figure 1).

### Difference by vascular malformation type

**Figure 2:**
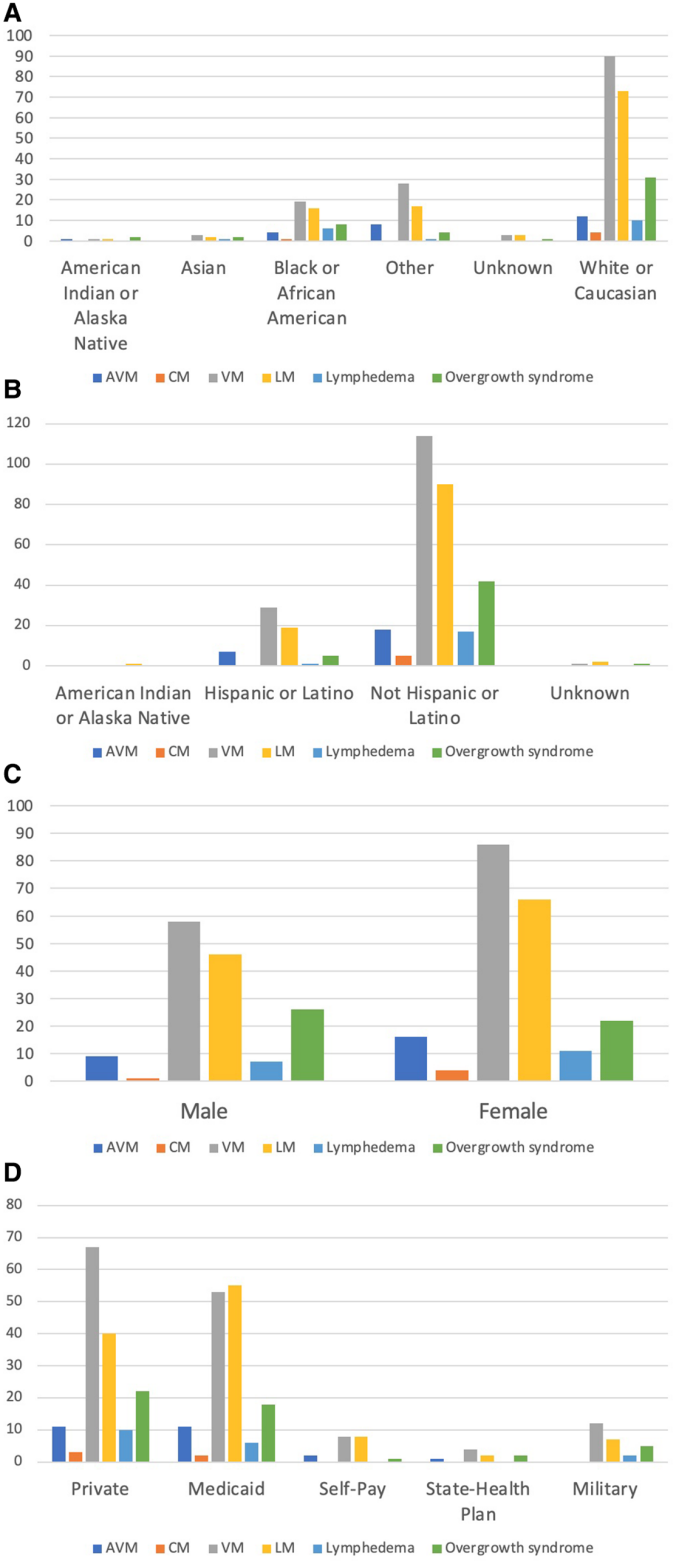
Comparison of vascular malformations by race, ethnicity, sex, and insurance. (**A**) Comparison of vascular malformations by race. (**B**) Comparison of vascular malformations by ethnicity. (**C**) Comparison of vascular malformations by sex. (**D**) Comparison of vascular malformations by insurance.

No significant difference was found when the various vascular malformations were compared by race (*χ*^2^(25, *N* = 352) = 22.90, *p* = 0.584), ethnicity (*χ*^2^(15, *N* = 352) = 10.85, *p* = 0.763), sex (*χ*^2^(5, *N* = 352) = 4.57, *p* = 0.471), and insurance status (*χ*^2^(20, *N* = 352) = 13.96, *p* = 0.833) (Figures 2A–D; Supplementary Tables I-IV).

**Figure 3:**
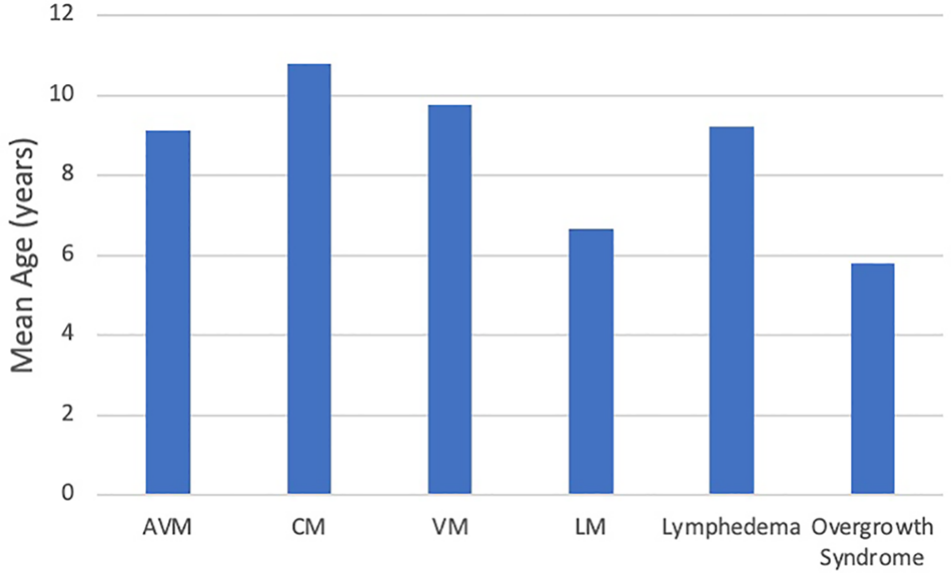
Age at presentation of vascular malformation.

When each vascular malformation was compared by age at presentation, an initial *p*-value of 0.0000176 indicated a difference existed, and further testing discovered that patients with VM were older at presentation than patients with both LM (mean 9.75 years vs. 6.66 years, *p* = 0.0022) and overgrowth syndrome (mean 9.75 years vs. 5.80 years; *p* = 0.0042) (Figure 3).

## Discussion

Health disparities have been extensively documented and are present within a vast number of diseases, affecting both disease incidence and outcomes. The existence and cause of disparities are not always intuitive, necessitating exploration of their existence and the ways they intersect with diseases. For example, in patients with hereditary hemorrhagic telangiectasia, white patients were found to have less pulmonary and cerebral AVMs compared to Asian and Hispanic patients respectively (10). In patients with diseases like vascular malformations where disparities are not extensively studied, research is needed to identify disparities where they exist so that these may be mitigated where possible.

There have been several studies examining sex differences among patients with vascular malformations. Some have found no difference similar to ours, while others observed females to be more likely to present with vascular malformation (11–13). Furthermore, multiple studies looking specifically at KTS have made conflicting claims about sex differences (14, 15). While literature does exist examining sex differences for vascular malformations, our study is the first in the literature to our knowledge to review several sociodemographic factors in pediatric patients with this diagnosis.

Vascular malformations are congenital malformations, some of which are caused by genetic mutations leading to impaired morphogenesis during development. The literature continues to grow with discoveries in genes responsible for some vascular malformations, but it is unclear if and which environmental factors also play a role in pathogenesis (16). Exploration of sociodemographic factors will help uncover the contribution of environmental factors beyond genetics, providing a more comprehensive understanding of vascular malformations. Many known causative genes in vascular malformations are somatic mutations whose expression may increase with environmental stressors, hinting at a potential role of environmental factors contributing to their development (16, 17). However, our findings demonstrated no difference in race, ethnicity, sex, and insurance status, indicating that the role of environmental factors in the development of vascular malformations may be less important than the role of genetics.

Patients with VM presented later than both LM and overgrowth syndromes which has not been previously reported. VM can cause a variety of symptoms depending on severity and location, ranging from cosmetic concerns to functional impairment, organ damage, thrombophlebitis, and increased risk of deep vein thrombosis (16, 18). Many VMs do not become symptomatic until adolescence or adulthood and are often misdiagnosed as a hemangioma which may indicate why there is a delay in their diagnosis (18, 19). LMs often present with symptoms of lymphedema, pain or swelling and most often occur around the head and neck (20). Overgrowth syndromes can be associated with vascular malformations or may be a component of certain syndromes. For example, Parkes-Weber syndrome commonly presents with CMs and overgrowth. However, patients often have AVMs which can have debilitating consequences (16). Mathes et al. described their findings that only 56.5% of vascular anomalies were diagnosed at birth (13). Their study also found that vascular malformations, in general, are misdiagnosed due to their complexity. This further supports the need for improving diagnostic accuracy among physicians so that early VM management can be initiated.

This study has several strengths, including a large cohort of patients with relatively rare diseases. This study also compares a wide variety of vascular malformations allowing for intergroup comparisons to be made, whereas existing literature often presents sociodemographic factors within the context of a specific condition.

As far as limitations, this was a single center study that primarily serves a single state. While this is helpful for providing epidemiological data for this region, it may limit the generalizability of the findings. In general, VM and LM are more common than isolated CM, and the disproportionate numbers within each group may prevent adequate comparison. Similarly, disproportionate group size in RUCA codes limited the ability to compare the number of patients per total population in each RUCA code. Categories created for simplicity led to the grouping of several heterogeneous conditions, such as “overgrowth syndrome” which contained several different syndromes with vastly different symptomology and etiology (16, 21). This potentially could have masked intra-group differences as well. Another limitation of this study is that it only tracked age at the presentation at a single vascular center which may be skewed if initial care was received at another center. While this study period was selected to include the most recent patient data, it included the onset of the Covid-19 pandemic. This may have altered the patient population that presented during this time if some patients delayed seeking care.

This study provides useful sociodemographic information about pediatric patients presenting with vascular malformations. Patients were primarily white, not Hispanic or Latino, female, had private health insurance, and were from the most urban setting. While no differences existed among the various vascular malformations for race, ethnicity, sex, and insurance status, patients with VM presented at a later age than patients with LM and overgrowth syndromes. This suggests that the development of these conditions *via* somatic mutations may be less affected by environmental factors and more by random genetic events. Improving accurate diagnosis upfront may bridge the gap of age presentation between VM and LM and overgrowth syndromes and improve care. Future research is needed to determine if sociodemographic factors like these studied lead to disparities in patient treatment and outcome.

## Data Availability

'The original contributions presented in the study are included in the article/Supplementary Materials, further inquiries can be directed to the corresponding author/s.
