# Supplementary material for: Sociodemographic characteristics of pediatric patients with vascular malformations: Results of a single site study
Source: Front Pediatr. 2023 Feb 16;11:1078611. doi: 10.3389/fped.2023.1078611 (PMC9978219; doi:10.3389/fped.2023.1078611)
Supplement: Supplementary file 1 [file Table1.docx]

Supplementary Table I. Comparison of Vascular Malformations by Race

|  | American Indian or Alaska Native | Asian | Black or African American | Other | Unknown | White or Caucasian | p-value= 0.584 |
| --- | --- | --- | --- | --- | --- | --- | --- |
| AVM | 1 | 0 | 4 | 8 | 0 | 12 |  |
| CM | 0 | 0 | 1 | 0 | 0 | 4 |  |
| VM | 1 | 3 | 19 | 28 | 3 | 90 |  |
| LM | 1 | 2 | 16 | 17 | 3 | 73 |  |
| Lymphedema | 0 | 1 | 6 | 1 | 0 | 10 |  |
| Overgrowth Syndrome | 2 | 2 | 8 | 4 | 1 | 31 |  |

Supplementary Table II. Comparison of Vascular Malformations by Ethnicity

|  | American Indian or Alaska Native | Hispanic or Latino | Not Hispanic or Latino | Unknown | p-value= 0.763 |
| --- | --- | --- | --- | --- | --- |
| AVM | 0 | 7 | 18 | 0 |  |
| CM | 0 | 0 | 5 | 0 |  |
| VM | 0 | 29 | 114 | 1 |  |
| LM | 1 | 19 | 90 | 2 |  |
| Lymphedema | 0 | 1 | 17 | 0 |  |
| Overgrowth Syndrome | 0 | 5 | 42 | 1 |  |

Supplementary Table III. Comparison of Vascular Malformations by Sex

|  | Male | Female | p-value= 0.471 |
| --- | --- | --- | --- |
| AVM | 9 | 16 |  |
| CM | 1 | 4 |  |
| VM | 58 | 86 |  |
| LM | 46 | 66 |  |
| Lymphedema | 7 | 11 |  |
| Overgrowth Syndrome | 26 | 22 |  |

Supplementary Table IV. Comparison of Vascular Malformations by Insurance Status

|  | Private | Medicaid | Self-Pay | State-Health Plan | Military | p-value= 0.833 |
| --- | --- | --- | --- | --- | --- | --- |
| AVM | 11 | 11 | 2 | 1 | 0 |  |
| CM | 3 | 2 | 0 | 0 | 0 |  |
| VM | 67 | 53 | 8 | 4 | 12 |  |
| LM | 40 | 55 | 8 | 2 | 7 |  |
| Lymphedema | 10 | 6 | 0 | 0 | 2 |  |
| Overgrowth Syndrome | 22 | 18 | 1 | 2 | 5 |  |
